# CO_2_-Based Polypropylene Carbonates with High-Stretch and Self-Healing Properties

**DOI:** 10.3390/ijms26083878

**Published:** 2025-04-19

**Authors:** Chiara Pasini, Stefano Pandini, Francesca Milocco, Jing Chen, Zhenchen Tang, Paolo P. Pescarmona, Luciana Sartore

**Affiliations:** 1 Department of Mechanical and Industrial Engineering, University of Brescia, via Branze 38, 25133 Brescia, Italy; chiara.pasini1@unibs.it (C.P.); luciana.sartore@unibs.it (L.S.); 2 Chemical Engineering Group, ENTEG, University of Groningen, Nijenborgh 3, 9747 AG Groningen, The Netherlands; miloccofrancesca@gmail.com (F.M.); jing.chen@rug.nl (J.C.); z.tang@njtech.edu.cn (Z.T.); p.p.pescarmona@rug.nl (P.P.P.)

**Keywords:** carbon capture and utilization, carbon dioxide-based copolymers, polypropylene carbonate, propylene oxide, heterogeneous catalysis, self-healing

## Abstract

Carbon dioxide-based copolymers such as polypropylene carbonate (PPC) can offer the double environmental benefit of capturing CO_2_ and replacing oil-based raw materials in the plastics industry with renewable ones. However, their production at an industrial level is still limited by the range of applications in which their physicochemical properties are competitive and ideally surpass those of fossil-based polymeric commodities. This work introduces PPC materials with high-stretch and self-healing properties that were prepared by copolymerization of CO_2_ and propylene oxide using tailored Zn glutarate catalysts. The PPC materials were analyzed in terms of composition, molecular weight, thermal and mechanical behavior, particularly focusing on their tensile properties, strain recovery, creep response, and self-healing ability. All the prepared PPC materials showed good ductility and self-healing properties. The most promising ones achieved excellent and fast recovery of extremely high elongations (>700%), still reaching remarkable values (>600%) after proper self-healing. These high-stretch and self-healing PPC materials are completely amorphous, present good optical transparency, and can be processed using techniques normally used for other thermoplastics. Therefore, they are promising for a variety of applications, including shrink films and self-healing packaging, thus providing new, valuable perspectives for the industrialization of these CO_2_-based polymers.

## 1. Introduction

One of the main challenges for a more sustainable plastics industry is the substitution of polymers derived from oil with polymers obtained from renewable raw materials. Plastics production has been continuously growing from 2 million tons in 1950 [1] to about 400 million tons in 2022, of which less than 10% is obtained in a circular economy perspective, i.e., from recycled plastics, bio-based plastics, or carbon capture, whereas the vast majority comes from non-renewable fossil-based sources that will eventually need replacement [2]. The largest technological hurdle is to address the transition towards renewable raw materials in a rapid and cost-effective way, maintaining, as much as possible, the existing production chains and avoiding repercussions on the performance of the final products. Among the proposed solutions, carbon dioxide-based copolymers have gained particular attention, since the use of CO_2_ has the double advantage of exploiting a renewable source and reducing the environmental impact of many industrial processes that release this well-known greenhouse gas as a byproduct [3]. Indeed, carbon dioxide is an abundant, non-toxic, and low-cost carbon source [4]. This has led to the development of a variety of strategies for carbon capture and utilization in the production of synthetic fuels, chemical feedstocks, and polymers, especially in the manufacture of urea, salicylic acid, polycarbonates, and polyurethanes [5].

Due to the high thermodynamic stability of CO_2_ molecules, copolymerization reactions require highly reactive comonomers, which makes epoxides particularly suitable, thanks to the significant amount of energy released during their ring-opening process (114 kJ/mol, ca.) [6]. When CO_2_ reacts with epoxides, it results in polyalkylene carbonates with valuable properties such as thermoplasticity, high Young’s modulus, high transparency, UV stability, gas barrier properties, and biocompatibility [7,8,9]. Therefore, these CO_2_-based polycarbonates have been studied for several applications, especially for the preparation of polymer products (polyols for the synthesis of polyurethanes, polymeric coatings, polymer blends and composites), for battery applications (solid polymer electrolytes and gel polymer electrolytes for lithium-ion batteries), and for biomedical applications (scaffolds for tissue engineering, controlled drug delivery, tumor imaging), but also as sacrificial binders in the production of ceramics and adhesives, as barrier materials and foams for packaging of food or medical supplies, as thin films for flexible electronics, and as non-woven fabrics [9,10,11,12].

Polypropylene carbonate (PPC) is the most studied CO_2_-based polyalkylene carbonate, introduced in 1969 by Inoue et al., who promoted the copolymerization of carbon dioxide and propylene oxide by means of ZnEt_2_-based catalysts [13]. Due to the very low reaction rate achieved with these and other dialkylzinc-based catalysts, research has been focusing on the development of new homogeneous and heterogeneous catalysts [12,14,15,16]. Furthermore, various studies were conducted to improve the performance of PPC, usually aiming at overcoming limitations in its mechanical strength at high temperatures and its thermal stability, or at reducing its brittleness at low temperatures, which are consequences of its relatively low glass transition temperature, typically close to room temperature. These goals were pursued mainly via terpolymerization, blending with other polymers, and addition of organic or inorganic fillers [8,11,17,18,19]. However, terpolymerization strategies are rarely economically affordable, while many polymer blends suffer from low miscibility, leading to weak intermolecular interactions between their components, so that plasticizing and toughening of PPC are seldom achieved simultaneously [20]. On the other hand, much less attention has been dedicated to the correlation between chemical/structural characteristics of PPCs and the mechanical and functional properties that may be useful in practical applications that are different from those of bisphenol-A-based polycarbonates. For example, for application in the packaging industry, it may be useful to investigate properties such as stiffness, strength, elongation at break, as well as shrink wrapping abilities, and to aim at materials displaying high-stretch and/or self-healing properties.

In particular, the development of self-healing polymers is inspired by the natural regenerative abilities of biological organisms, which possess built-in repair mechanisms to regain their functions after physical damage [21,22]. This feature could contribute to the application of PPC in the field of smart materials, for example, in flexible and wearable electronic devices [23] or as scratch self-healing material [24]. At the same time, such smart devices or packaging solutions would gain in sustainability, thanks to the improved durability that may be achieved through self-repair [25,26]. Many possible mechanisms for self-healing have been explored, mostly related to interdiffusion of polymer chains, shape-memory-assisted approaches, formation of reversible bonds by chemical reactions or supramolecular interactions, or extrinsic mechanisms based on self-healing agents embedded in capsules or vessels [21,27,28]. It is worth noting that PPC has shown self-repair abilities when physically crosslinked with a network of cellulose microfibers, forming a composite with shape memory properties [24], but to the best of our knowledge, no studies have investigated this behavior in pure, unmodified PPC.

In this study, a set of PPC materials was prepared through a heterogeneous catalytic copolymerization reaction from CO_2_ and propylene oxide in the presence of novel zinc-based catalysts, without the addition of plasticizers or other modifications. The synthesized materials were analyzed in terms of composition, molecular weight, thermal properties, and mechanical behavior. The viscoelastic response of the PPC materials was investigated by tensile tests conducted at different strain rates and by multi-temperature creep tests, highlighting the effects of time and temperature on the mechanical properties. The study highlighted specific beneficial features of the obtained materials, such as rapid strain recovery of high elongations, and self-healing capability, through which two PPC surfaces can adhere and create a sealing or repair a defect.

## 2. Results

### 2.1. Synthesis of Polypropylene Carbonate (PPC) Materials

**Figure 1:**
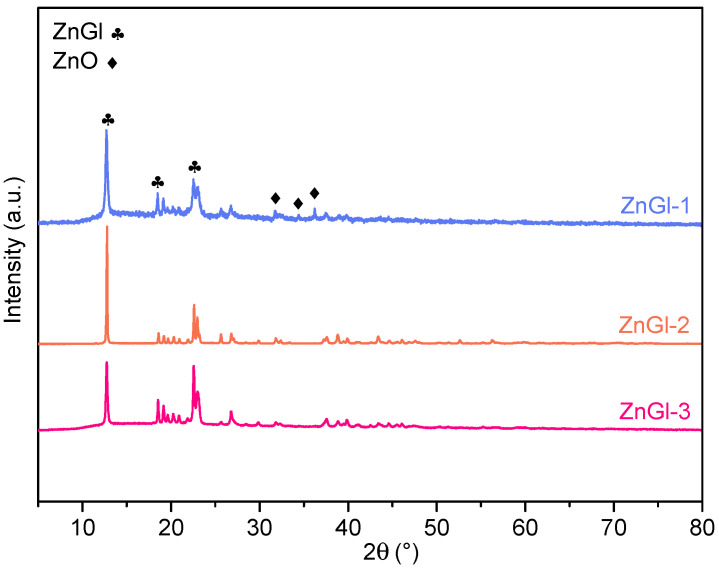
XRD patterns of the zinc glutarate catalysts used to prepare the polypropylene carbonate (PPC) materials.

A set of five polypropylene carbonate (PPC) materials was synthesized through the copolymerization of CO_2_ and propylene oxide in the presence of a zinc glutarate catalyst in a batch reactor at 80 °C and 40 bar CO_2_ for 20 h. Three zinc glutarate catalysts were utilized: the first two are based on a patented procedure developed by the authors [29] (with the two catalysts, ZnGl-1 and ZnGl-2, differing slightly in the nature of the zinc precursor from which they were prepared) and the other was inspired by the literature [30] (ZnGl-3). Characterization of the three catalysts by XRD indicated that they all present the diffraction pattern of zinc glutarate, though ZnGl-1 also contained a small fraction of unreacted ZnO (Figure 1). The highest activity in the synthesis of PPC was achieved with one of the catalysts inspired by the patent procedure (ZnGl-1, reaching a productivity = 2.17 g_PPC_/(g_cat_∙h), compared to 1.86 g_PPC_/(g_cat_∙h) for ZnGl-3 under the same conditions). However, rather than on the catalytic performance, the focus of this work is on the properties of the prepared PPC materials. In this context, we chose to test different zinc glutarate catalysts to investigate to what extent their features would affect the properties of the obtained polymers. Our results show that the nature of the zinc glutarate catalyst is not the main factor determining the differences in physicochemical and thermal properties observed among the produced PPC materials (compare the data in Table 1 for the three polymers prepared with ZnGl-1 to those for the polymers synthesized with ZnGl-2 and ZnGl-3). On the other hand, we observed that, as a function of the storage time of the catalyst and of the propylene oxide used as reactant, the presence of adventitious water (in the catalyst or in the epoxide), the scale of the reaction, and the purification method, the properties of the PPC materials can differ in terms of content of residual cyclic propylene carbonate (CPC) by-product, percentage of ether linkages in the polycarbonate backbone, and molecular weight (weight-average molecular weight, M_w_; number-average molecular weight, M_n_; polydispersity index, D) (see Table 1) [30]. It is worth noting that the prepared products contain a significant fraction of CPC, which can act as a plasticizer and thus have a relevant effect on the properties of the polymers, in particular with regard to the stiffness at room temperature and the glass transition temperature [31,32]. All the PPC materials in this work were purified to remove CPC and the catalyst. It was observed that it is possible to minimize the residual CPC content after purification by pouring the reaction mixture more slowly into the HCl/MeOH solution (see Section 3. “Methods”) and by cutting the purified polymer into small pieces and sonicating it repeatedly in MeOH. With these optimized conditions, a polymer with high purity (>99.5%, as determined by ^1^H NMR) was obtained in the materials labelled as PPC100 and PPC103.

Since the purpose of this work is to explore the stretch and self-healing behavior of the PPC materials and to investigate how these features are influenced by their physicochemical properties, the catalytic results will not be discussed further, and the focus will be entirely on the properties of the five PPC materials.

The prepared PPCs differ not only in terms of residual CPC content but also of the weight-average molecular weight (M_w_), which ranges between 45·10^3^ and 353·10^3^ g/mol, and of the number-average molecular weight (M_n_), which ranges between 14·10^3^ and 59·10^3^, with similar values of polydispersity index (D) for all systems. The obtained PPC macromolecules are composed of more than 90% carbonate linkages and a lesser amount of ether linkages, ranging overall between 5% and 7%. The higher the carbonate linkage content, the larger the fraction of CO_2_ entrapped in the polymer.

### 2.2. Thermal Properties

**Figure 2:**
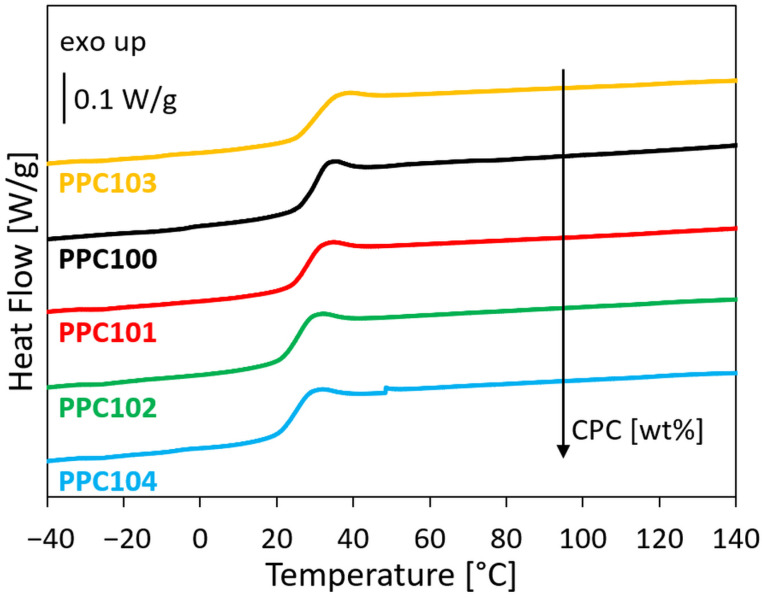
DSC traces of the PPC materials (second heating scans) were ordered on the basis of the CPC content.

Thermal characterization of the materials by DSC and TGA was carried out in order to investigate their thermal behavior, especially identifying those temperatures at which they undergo glass transition and thermal degradation phenomena. DSC second heating scans are reported in Figure 2, and the values of the glass transition temperature (T_g_) and degradation temperature (T_deg_) are shown in Table 1.

The DSC traces show that all PPC batches are amorphous, as confirmed by the absence of any endothermal signal that could be linked to a crystalline phase, and allow evaluating their T_g_, which corresponds to the inflections visible in the second heating scans. Small but clear differences in the glass transition temperatures are observed, between about 25 °C and 30 °C. These differences may be correlated with the material composition, in particular with small variations in the CPC content and in the percentage of ether linkages in the PPC macromolecules. Indeed, CPC molecules have been reported to exert a plasticizing effect [31,32], and the presence of ether linkages is known to improve the flexibility of the polymer chains, lowering the T_g_ [33]. However, the percentage of ether linkages is relatively similar in the prepared PPC materials (between 4.9 and 6.5%, see Table 1), and no clear correlation is found between the T_g_ values and the ether linkages % (see Figure S2b). On the other hand, a good correlation is observed between the T_g_ values and the CPC content (Figure 2 and Figure S2a). This suggests that in the explored range, the CPC content plays a more relevant role than the percentage of ether linkages in defining the T_g_ value of the PPCs. It is worth noting that, based on the literature, the molecular weight of PPC materials also has an influence on their T_g_, with higher molecular weights leading to higher glass transition temperatures [34]. However, this effect was reported to be noticeable for low-molecular-weight PPCs, but to flatten out for high-molecular-weight PPCs (typically at M_n_ > 15,000 g/mol [34]), as those reported here. Therefore, the differences in M_w_ and M_n_ between the PPC materials reported here are unlikely to have contributed significantly to the observed differences in T_g_ values.

Further information about the thermal behavior of the PPC materials is obtained by TGA, which reveals that these materials are thermally stable until temperature values of about 200–250 °C (T_deg_, reported in Table 1 and depending on the specific PPC considered), in correspondence of which they undergo a major degradation (see TGA traces in Figure S3). A factor that may influence the thermal degradation behavior is the amount of ether linkages, which have been reported to increase the values of T_deg_ because ether bonds are more stable than carbonate bonds [33]. Overall, systems with lower percentage of ether linkages (PPC100, PPC101 and PPC102) show lower thermal stability (T_deg_ around 195–210 °C), while the polycarbonates that have a larger fraction of ether linkages (PPC103 and PPC104) display higher values of T_deg_ (225–245 °C) (see Table 1).

### 2.3. Tensile Properties

**Figure 3:**
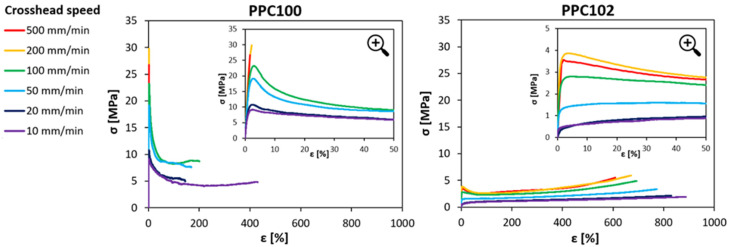
Examples of stress-strain curves under tensile conditions at various strain rates for two different polycarbonates (PPC100 and PPC102). The inserts in the graphs report magnifications of the curves for strain values up to 50%.

The mechanical behavior of the PPC materials was characterized at room temperature under tensile conditions. Since thermal analyses revealed that the PPC materials undergo a glass transition close to room temperature, the implications of the material’s viscoelastic response were regarded with special consideration. Therefore, the effect of the strain rate on the mechanical properties was explored by carrying out the tests at different crosshead speeds, between 10 mm/min and 500 mm/min (corresponding to strain rates between 2.8·10^−3^ s^−1^ and 1.4·10^−1^ s^−1^). Examples of tensile stress-strain curves at various strain rates are reported in Figure 3 for two PPC samples selected to show the most evident differences in mechanical behavior, one appearing as the most ductile (PPC102) and the other one being the least ductile (PPC100). Three types of behavior can be observed:

(i) Ductile response with smooth change of slope at yielding: In this case, the curves display a nonlinear increase in stress at small strains, followed by a transition to a lower slope. This behavior is mainly found in polycarbonates having lower T_g_ (such as PPC102 and PPC104), especially when tested at low strain rates.

(ii) Ductile response with relevant strain softening at yielding: in this case, the stress increases almost linearly up to yield, when it drops significantly and then starts increasing again, but with a much lower slope. This is observed both in polycarbonates with higher T_g_ (PPC100, PPC101, PPC103) tested with low crosshead speed, and in polycarbonates with lower T_g_ (PPC102, PPC104) tested with higher crosshead speed.

(iii) Brittle failure: In this case, the curves exhibit a linear increase in stress until sudden failure of the specimens at small strains. This occurs only at high strain rates in polycarbonates with higher T_g_ (such as PPC100 and PPC101).

**Figure 4:**
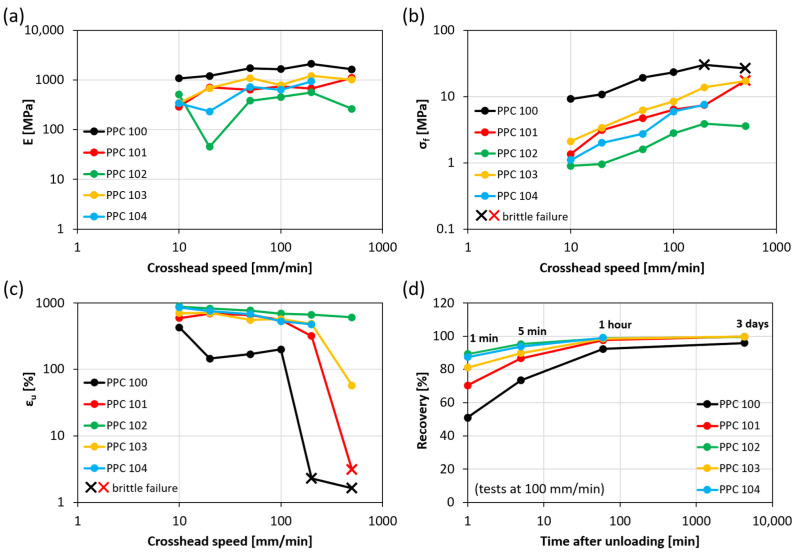
Young’s modulus (**a**), failure stress (**b**), ultimate tensile strain (**c**), and strain recovery (**d**) for various PPC batches and various crosshead speed values. Strain recovery is reported over time for a crosshead speed of 100 mm/min.

A more complete description of the mechanical response of all the obtained materials under all strain rate conditions is given in terms of Young’s modulus (E), failure stress (σ_f_), and ultimate tensile strain (ε_u_) (Figure 4a, Figure 4b, Figure 4c, respectively). These results are plotted against the crosshead speed on a logarithmic scale to highlight the effect of the strain rate on the mechanical properties. As the crosshead speed increases, stiffness and strength improve, while the strain at break tends to decrease.

More in detail, Young’s modulus values are mostly within 200 MPa and 2000 MPa and show both a dependence on the PPC material considered and a moderately increasing trend as the crosshead speed grows, such that E values measured at high strain rates are on average 2–3 times higher than those obtained at low strain rates. Failure stress values display higher variability, up to one order of magnitude for the same material batch, and overall range between about 1 MPa and 30 MPa. When the response is ductile (above-mentioned behaviors i and ii), the failure stress corresponds to the yield stress, measured in correspondence with the first change in the slope of stress-strain curves. Conversely, for specimens exhibiting brittle failure (behavior iii), σ_f_ represents the stress at break, denoted by a cross in the graph in Figure 4b. Finally, ultimate tensile strain values are typically very high for strain rates within 100 mm/min (over 500%, except for PPC100, presenting ε_f_ between 150% and 450%), which is achieved without any modification after the synthesis, unlike other PPCs with enhanced ductility obtained via blending with low molecular weight plasticizers or other polymers [3,20]. The ultimate tensile strain decreases slightly with the crosshead speed until, at high strain rates, a sharp brittle transition causes a reduction of ε_f_ down to 2–3% (data points marked with a cross in Figure 4c). The more ductile the PPC material, the higher is the minimum crosshead speed required for brittle failure (200 mm/min for PPC100; 500 mm/min for PPC101; higher than the tested speed values for the other batches).

The literature regarding CO_2_-derived PPCs shows a high variability of data in terms of stiffness, strength, and elongation at break, due to differences concerning several aspects, ranging from the synthesis conditions and the molecular weights to mechanical test parameters (see Table 2, where our results are reported together with the outcomes of several other studies [3,11,18,19,35,36,37,38]). Such a wide range of results and settings limits the possibility of making direct comparisons of the tensile properties presented here with the data from other studies. Nonetheless, the broad range of values found for the PPCs in this study covers the large majority of the intervals reported in the literature (Table 2). It may be noted that moderately higher tensile strength (around 40 MPa) was achieved for some PPCs in the literature [3,38], but this was obtained only for systems with very low strain at break (4–7%). Most importantly, the materials presented here stand out for their very high stretchability, especially when taking into account at least the crosshead speed at which the tests are conducted. In particular, the crosshead speed for the works in Table 2 (when reported) was always between 2 mm/min and 50 mm/min, corresponding to elongations of 600% or higher for all the investigated PPCs except PPC100; comparable values were found only in the paper by Trofimchuk et al. [38], and only for crosshead speeds within 10 mm/min.

Interestingly, the variability encountered in the mechanical response of the PPC batches can be correlated here with their T_g_, which is likely influenced by the presence of CPC in the materials, as discussed in Section 2.2. In particular, PPC100, PPC101, and PPC103 present higher values of T_g_ (28–30 °C), so they tend to be stiffer/stronger and less ductile, switching from ductile to brittle behavior at high strain rates. These characteristics are especially pronounced in PPC100. On the other hand, PPC102 and PPC104 have lower T_g_ (25–26 °C), which is likely the reason for their improved ductility. Indeed, these materials clearly manifested yielding at all strain rates, with yield stress not exceeding a few MPa and limited (or totally absent) subsequent strain softening.

In addition, the ability of PPC to recover strain at unloading can be observed in Figure 4d, reporting strain recovery as a function of time after the end of tensile tests carried out with a crosshead speed of 100 mm/min. The materials with lower T_g_ (PPC102 and PPC104) can revert to their original length particularly fast, showing values of recovery of 87–89% after just one minute from unloading, when those with higher T_g_ (PPC 100, PPC101 and PPC103) are still significantly behind (recovery values of about 50%, 70%, and 80%, respectively). This may be related to the higher mobility of the macromolecules in systems with lower T_g_. Despite these differences at short times, all PPC batches exhibit extraordinary recovery of the large strains reached during tensile tests (ε_f_) within 1 h (recovery equal to 98–99%), except for PPC100 (recovery equal to 92%). The latter reaches about 96% recovery after a few days, when the other batches have fully restored their initial length.

Notably, the mechanical characterization revealed that these materials may find successful application in the packaging field, thanks to their adequate stiffness as well as their ease at being plastically deformed, their high stretchability, and their ability to quickly and fully recover deformation after being stretched up to exceptionally high strains. In addition, the possibility of tuning their properties depending on the strain rate allows for different applications, such as films for manual wrapping (low strain rates) or for industrial wrapping (high strain rates).

### 2.4. Creep Behavior

Multi-temperature creep tests were carried out in order to further investigate the viscoelastic response of the PPC samples and to gain a deeper understanding of the effects of time and temperature on the material behavior. The analysis was carried out by building the creep compliance master curve of each material through the application of a time-temperature superposition scheme. For a given reference temperature, these master curves allow to identify more clearly the different regions in the viscoelastic response and, in particular, two important characteristic times, i.e., the retardation time (t_ret_), which is associated to the main relaxation process occurring at T_g_, and the flow time (t_flow_), at which the material enters the viscous flow regime.

**Figure 5:**
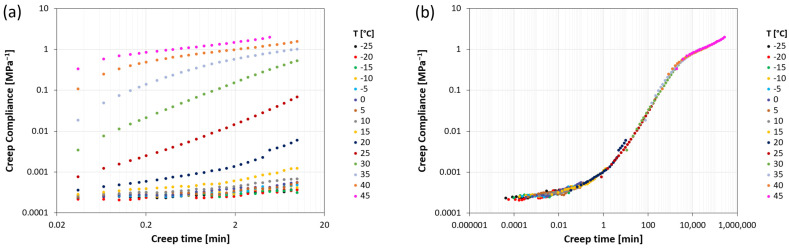
(**a**) Isothermal creep compliance curves at various temperatures for PPC104; (**b**) creep compliance master curve representation obtained by superposition of the isothermal curves at a reference temperature T_0_ = 20 °C.

Creep compliance curves for the various systems were obtained starting from isothermal creep curves, which were mutually shifted according to the time-temperature equivalence principle, as shown as an example for system PPC104, in Figure 5. Isothermal creep curves (Figure 5a) were measured on an experimental window of 10 min and for temperatures equally spaced by 5 °C between −25 °C and the temperature at which, due to the onset of flow, the elongation reaches the maximum extension allowed in the testing instrument. The resulting curves show an increasing trend of creep compliance with time, occurring at different rates depending on the temperature. The isothermal curves present a good degree of similarity, allowing them to be shifted along the time scale until superposition to form a complete master curve, for a given reference temperature. The result is displayed in Figure 5b for PPC104 at a reference temperature T_0_ = 20 °C. This curve shows the whole evolution of creep compliance with time, starting with the lowest values in the glassy plateau, significantly increasing along the glass transition region, and suggesting a rubbery plateau at higher values before entering the flow regime. From the curve, it is possible to evaluate the retardation time (t_ret_), close to the inflection point of the sigmoidal trend, and the flow time (t_flow_), here taken as the last instant of test due to the above-mentioned elongation measurement limit. For example, Figure 5b shows that, for PPC104 at 20 °C, the time scale of the retardation process is around 100 min, whereas that of the entrance in the flow regime is higher than 10^5^ s.

**Figure 6:**
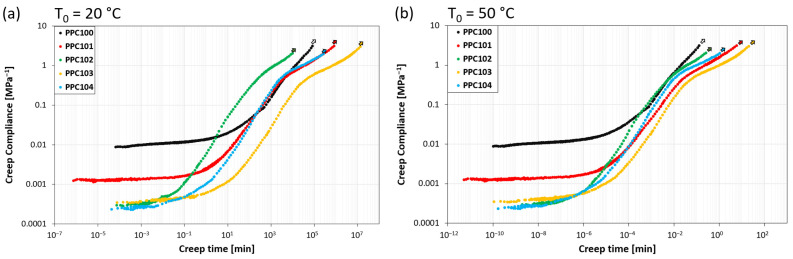
Creep compliance master curves for all PPC systems at reference temperature (**a**) T_0_ = 20 °C and (**b**) T_0_ = 50 °C.

By building the master curves of all the investigated systems, the same behavior found for PPC104 can be observed, but with different values of retardation time and flow time. All the master curves are reported in Figure 6a for T_0_ = 20 °C and Figure 6b for T_0_ = 50 °C, and the corresponding values of t_ret_ and t_flow_ are summarized in Table 3. While the data at T_0_ = 20 °C are representative of the response at room temperature, which is close to the glass transition, those at T_0_ = 50 °C describe the behavior well above T_g_, more specifically at the highest temperature applied during self-healing experiments.

The compliance master curves at T_0_ = 20 °C show variations in the viscoelastic behavior depending on the specific polycarbonate considered. It is noteworthy that the transition between the glassy and rubbery plateaus occurs at different retardation times, varying between about 10^0^ and 10^3^ min. PPC102 shows the shortest t_ret_ (about 3 min), followed by PPC104 and PPC101 (ca. 10^2^ min, i.e., about 0.1 days), and finally by PPC103 and PPC100 (10^3^ min, i.e., about 1 day). The results show that the retardation time increases with the glass transition temperature of the specific PPC. This is not surprising, since the materials share a very similar structure and thus, supposedly, their viscosity has a similar decreasing dependence on temperature. Therefore, the higher the glass transition temperature is with respect to the reference temperature, the longer is the time required to approach retardation/relaxation processes [40]. The entrance in the flow regime also varies, with values of flow time between about 10^4^ and 10^7^ min and a trend among the different PPC materials similar to the one observed for the t_ret_ values. The only change in the sequence is that PPC100 has the longest retardation time but the second shortest flow time, since it presents a particularly short rubbery plateau, with an almost immediate entrance in the flow regime.

By looking at the compliance master curves at the highest reference temperature (T_0_ = 50 °C, well above the T_g_ of all materials), retardation times and flow times become shorter of about five or six orders of magnitude, so that phenomena occurring over days at T_0_ = 20 °C take place on the time scale of seconds at T_0_ = 50 °C, owing to the thermally-promoted increase in the macromolecular mobility [40]. Retardation times display the same ranking observed at 20 °C but become all shorter than 1 s. This suggests that, for most practical applications, regarding time scales above 1 s, the material response at this temperature is dictated by the rubbery-like and flow behavior. While no significant differences among the systems are visible in the rubbery plateau, the most striking difference is found for the entrance in the flow regime. In particular, PPC100 displays the earliest entrance in this regime (t_flow_ in the range of seconds), followed at slightly longer timescales by PPC102 and PPC104 (t_flow_ still within 1 min), and finally by PPC101 and PPC103, the latter being the one with the longest flow time (about 20 min). The early entrance of PPC100 in the flow regime may be related to its molecular weight being the lowest (Table 1) [41,42].

### 2.5. Self-Healing Behavior

At the macroscopic level, the PPC films exhibited a noticeable degree of tackiness and the capacity to bond with one another without readily detaching, indicating a potential for self-healing. This behavior manifested itself to differing extents based on the material composition. In this context, this work investigated the feasibility of repairing a film that has failed due to tearing or perforations by overlapping the edges around the damage. The self-healed area was created by partially overlapping, for a certain length, two strips of each PPC material, with or without exerting additional pressure. Then, the self-healed specimens were subjected to two separate tests: (i) single-lap shear tests, to determine if the self-healed joint between PPC surfaces can withstand the shear stress that originates under tension; (ii) T-peel tests, to assess whether the self-healed materials resist delamination. These tests helped identify the materials and the joining conditions that lead to an effective self-healing behavior.

#### 2.5.1. Self-Healing in Single-Lap Joint Configuration

Single-lap joint specimens were prepared and tested as described in detail in the “Methods” section (Section 3.2.7), by cutting long strips into two halves, overlapping the two halves for 5 mm, and subjecting them to tensile tests. These tests aimed at measuring the ability of the material to support the shear stress generated by a tensile load without failing in the repaired zone. To assess the time required for efficient self-healing, the tests were carried out at various times after overlapping their extremities, ranging from 10 min to 1 week. Moreover, self-healing was investigated under the effect of various environmental conditions: to achieve self-healing, the specimens were maintained either at room temperature (T_room_) or at 50 °C, and the overlapped extremities were simply put in contact (applied pressure: 0 MPa) or pressed together under an additional load (applied pressure: 0.3 MPa). All testing conditions are summarized in Section 3.2.7.

**Figure 7:**
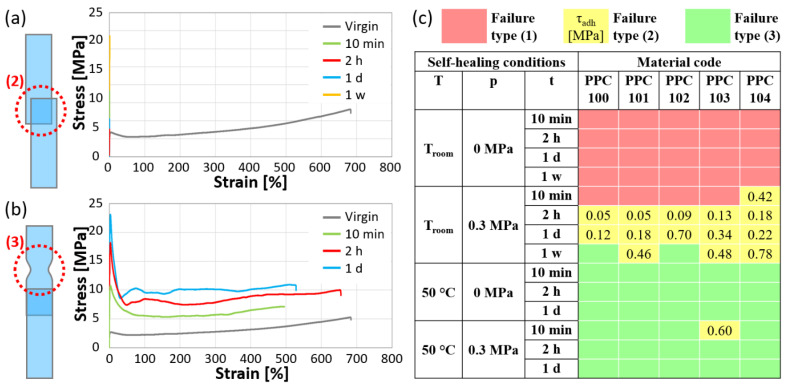
Examples of stress-strain curves obtained by single-lap shear tests after different self-healing times: (**a**) tests ending with failure type (2), i.e., detachment of the overlapped layers (PPC104 self-healed at T_room_ and 0.3 MPa); (**b**) tests ending with failure type (3), i.e., effective self-healing and yielding outside of the overlapped region (PPC104 self-healed at 50 °C and 0 MPa). Each set of curves is accompanied by a schematic representation of the failure type and compared with the stress-strain curve of virgin PPC104 for the same crosshead speed of single-lap shear tests. (**c**) Maps of the failure type for specimens joined under various conditions of temperature (T), pressure (p), and time (t) [red: type (1), i.e., no attachment of the two surfaces; yellow: type (2), with indication of the adhesive strength τ_adh_, in MPa; green: type (3)].

The test results can be categorized in three scenarios: (1) the overlapped surfaces do not adhere together; (2) the overlapped surfaces adhere together but undergo adhesive failure under the applied stress; (3) the overlapped surfaces adhere together and the joint exhibits superior integrity compared to the rest of the specimen, ultimately failing under tension by yielding in a region outside the self-healed area. Only in this latter case can a proper self-healing be claimed, suggesting the occurrence of good cohesion between the two surfaces, which is sufficient to avoid detachment under tensile stress and lead to failure by yielding elsewhere. In scenario (1), the specimens could not be tested under tension, whereas specimens that displayed failure types (2) and (3) exhibited distinct characteristics in the stress vs. strain correlations. Figure 7a and Figure 7b provide examples of single-lap shear test results obtained from the same system (PPC104) under varying self-healing settings, ending with failure types (2) and (3), respectively. The curves were obtained by calculating the stress values as if the specimens had a constant thickness equal to the pristine PPC strips. In particular, Figure 7a shows stress-strain curves of PPC104 after joining the surfaces at T_room_ and 0.3 MPa for various times, compared with the curve of virgin PPC104 tested with the same crosshead speed. The curves of these single-lap joint specimens appear as straight lines suddenly interrupted at low strains (<1%) because of the ineffective adhesion at the joint, leading to failure type (2). On the other hand, Figure 7b represents cases in which PPC104 showed failure type (3) after joining at 50 °C and 0 MPa at various times. All these curves show the achievement of a ductile response with ultimate strain values comparable with those of the virgin material.

The conditions promoting the various types of failure are illustrated in Figure 7c with a color code: red, for failure type (1); yellow, for failure type (2); green, for failure type (3). In addition, for the tests with failure type (2) (i.e., the only ones showing adhesive failure), information about the adhesion strength of the two overlapping surfaces is reported as τ_adh_ in the figure (see Equation (2) in Section 3.2.7).

At room temperature (ca. 23 °C), the self-healing was slow and mostly ineffective, as no significant adhesion was detected without the application of pressure, even after one week, precluding any testing [failure type (1)]. Applying a pressure of 0.3 MPa promoted adhesion, although more than 10 min were still required for the two surfaces to adhere (except for PPC104). Failure mostly occurred at the interface [failure type (2)]. Notably, the adhesion strength, τ_adh_, tends to increase with time under these self-healing conditions. After one week at a 0.3 MPa pressure, two PPCs, PPC100 and PPC102, even showed full self-healing [failure type (3)]. It is worth noting that the retardation times associated with the glass transition during creep tests were never found to be sufficient for effective self-healing and that the application of a small pressure guaranteed complete self-healing only for the two materials with the shortest flow times at T_0_ = 20 °C (60 days for PPC100; 7 days for PPC102).

Much more efficient self-healing can be obtained by increasing the temperature at which the specimens are allowed to self-heal to 50 °C, which is above the glass transition of all PPC materials (see Table 1). For the PPC materials self-healed at 50 °C at both pressure conditions, the specimens showed strong cohesion between the overlapped surfaces, and this was almost always sufficient to prevent peeling at the self-healed joint, leading to failure by yielding elsewhere. In particular, proper self-healing required less than 10 min, and it is interesting to observe that such a time scale would allow the PPCs to enter a regime of flow or very close to it, as the flow times at this temperature are considerably short (ranging from 0.26 to 20 min).

#### 2.5.2. Self-Healing in T-Peel Configuration

The resistance of the self-healed specimens to peeling was studied by T-peel tests, in a configuration similar to that typically employed in the characterization of adhesive joints [43], as described in detail in Section 3.2.7. These tests were conducted by first joining two strips of material for most of their length and then trying to progressively separate them by pulling apart the two non-adhered extremities. Similarly to the approach used in the single-lap shear tests, the specimens were allowed to self-heal for various times before peeling (between 10 min and 1 day), at room temperature (T_room_) or at 50 °C, and either without any applied pressure (0 MPa) or under a constant pressure of 0.03 MPa, as summarized in Section 3.2.7. Also, this type of test presents three potential failure scenarios: (1) the overlapped surfaces do not adhere together, so that specimens cannot be tested; (2) the overlapped surfaces adhere together and failure occurs by delamination; (3) the overlapped surfaces adhere together in such a strong way that the self-healed region does not even participate to the failure process, which then occurs by tensile deformation of the gripped extremities.

**Figure 8:**
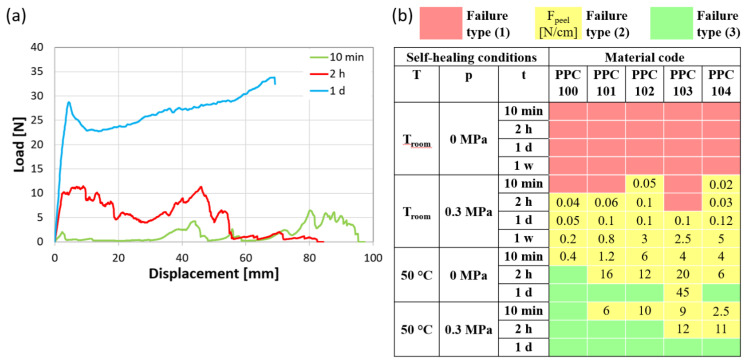
(**a**) Examples of load-displacement curves obtained by T-peel tests (PPC102 self-healed at 50 °C and 0 MPa for different times), ending with adhesive failure (10 min; 2 h) or with failure outside the self-healed area (1 d). (**b**) Maps of the failure type for specimens joined under various conditions of temperature (T), pressure (p), and time (t) [red: no attachment of the two surfaces (1); yellow: failure of the overlapped layers by peeling (2), with indication of the peeling force per unit width, F_peel_, in N/cm; green: effective self-healing and failure outside of the overlapped layers (3)].

Figure 8a illustrates the characteristic load vs. displacement curves for T-peel specimens with failure types (2) and (3), considering as an example PPC102 self-healed at 50 °C without pressure for different durations (10 min, 2 h, 1 d). Specimens joined for 10 min and 2 h are representative of failure type (2). Following an initial increase in load, during which the specimens’ non-adhered extremities are fully extended, the peeling process begins. This part of the curve is characterized by a force plateau with significant force oscillations due to the intermittent nature of fracture propagation, involving cycles of crack growth followed by subsequent cessation. This phenomenon is commonly termed “stick-slip” regime and arises from the interplay between the variations in driving force and the alterations in crack growth resistance [44]. By analyzing the force exerted during peeling, the level of adhesion may be assessed. The force necessary to peel a unit-width specimen, F_peel_, was evaluated by Equation 3 (Section 3.2.7).

In contrast, in the event of complete self-healing (e.g., load-displacement curve of PPC102 maintained for 1 day at 50 °C and 0 MPa in Figure 8a), failure arises from the deformation under tension of the specimen’s gripped extremities, resulting in a continuous increasing trend until load drop, initially due to the detachment of the paper reinforcement and then culminating in sudden tensile failure. This failure testifies to the achievement of an intimate cohesion between PPC surfaces and represents the attainment of proper self-healing in this type of test [45,46].

Figure 8b presents a map that summarizes the T-peel test results for the different PPC systems under each set of self-healing conditions, utilizing a color code to indicate the type of failure [red for failure type (1), yellow for failure type (2), and green for failure type (3)], and indicating the peeling force per unit width when adhesive failure occurred.

In accordance with the results of single-lap shear testing, self-healing at room temperature was slow and occasionally insufficient, resulting in all specimens either failing to bond or exhibiting peeling failure. Strips overlapped at room temperature without applied pressure did not adhere, and this also occurred for some specimen joints subjected to pressure for the shortest times. The other specimens joined at room temperature exhibited sufficient adhesion to be tested and underwent peeling. As expected, the peeling strength increased over self-healing time, as shown by the measured values of F_peel_ (Figure 8b) [45,47]. For instance, for most of the materials, F_peel_ varied from around 0.1 N/cm after 1 day of self-healing to about 1 N/cm or higher after 1 week.

By treating the specimens above their T_g_, the peeling force for a given self-healing time increased, until full self-healing was finally achieved after a specific time that depends on the material and on the presence or absence of applied pressure. It should be noted that increasing the temperature above T_g_ (or lowering the T_g_) promotes diffusion and conformational changes in thermoplastics by enhancing segmental chain mobility. This favors, though does not ensure, a successful self-healing by chain interdiffusion [21]. In this study, in the absence of pressure, most of the specimens achieved complete self-healing for treatments of 1 day. The required time became shorter under a pressure of 0.03 MPa, which led to efficient self-healing within 1 day for all the specimens, and even after within 2 h for some of them.

It is important to highlight that effective self-healing in T-peel tests requires longer times with respect to those found for single-lap shear tests. Full self-healing of T-peel specimens was never attained at T_room_, and always required longer self-healing times at 50 °C, typically 2–3 orders of magnitude longer than the corresponding t_flow_. More specifically, less than 10 min at 50 °C were sufficient to obtain self-healing of almost all single-lap joint specimens, whereas T-peel specimens required variable times from less than 10 min to more than 1 day, depending on the application of pressure and on the specific material. This is due to the fact that the adhered surfaces are more easily separated by opening forces that peel them apart than detached by shear forces. Thus, to withstand peeling, a more intimate cohesion was found to be needed.

The mechanisms of molecular diffusion at the basis of self-adhesion in self-healing thermoplastics may involve chain entanglement and interpenetration of the molecules belonging to each side of the overlapped region [28,48]. According to Mhlanga and Mphahlele [28], the merging of two polymer surfaces would follow a series of events, from surface rearrangement through surface approach (i.e., contact of the damaged or fractured sites by a 10 nm order of closeness), to wetting, and finally to diffusion. During these phases, rearrangements enabled by molecular mobility gradually reduce the distance between the two surfaces and replace the compromised interface with a new region formed by entangled polymer chains, becoming progressively stronger as diffusion continues and the entanglement density in this zone increases. A detailed explanation of the processes at the basis of molecular diffusion is provided by Voyutskii and Vakula [48], who ascribe the process to the cooperative motion of so-called kinetic units of linear macromolecules, i.e., segments of 20–30 carbon atoms capable of moving to new equilibrium positions, in particular when they find themselves close to microvoids. According to this interpretation, as the temperature rises, more microvoids appear by thermal expansion, and the kinetic energy of the system increases, until the sum of the activation energies for the individual chain segments overcomes the energy barrier for the diffusion of the whole macromolecule.

Finally, Table S1 helps summarize the aforementioned considerations on the times required for self-healing, comparing them with the times characteristic of different chain motions of the PPCs found during the creep tests (paragraph 2.4, t_ret_ and t_flow_). Similarly to what observed here, also other studies highlighted the importance of molecular relaxation and flow times in self-healing polymers, including the relaxation time of supramolecular structures, that of chain segments involved in the glass transition (which is here associated to t_ret_), and the time for mutual flow of the polymer chains (here denoted as t_flow_) [49,50]. In particular, the time to achieve proper self-healing has been correlated in the literature with the longest among the characteristic times related to the flow or rheological behavior of the polymer. This is consistent with our work, which suggests that for each PPC, a proper self-healing may be achieved for time scales comparable to (or larger than) the longest of its characteristic times (i.e., t_flow_). More specifically, the experiments on single-lap joint specimens showed sufficient self-healing for times close to t_flow_ or even shorter, i.e., when just approaching the flow regime or at its beginning, whereas the times to attain proper adhesion in T-peel specimens were around 2–3 orders of magnitude longer, i.e., after the flow regime was well developed.

## 3. Methods

### 3.1. Preparation of the Zinc Glutarate Catalysts

Three zinc glutarate catalysts were used in this work (Table 1). ZnGl-1 and ZnGl-2 were prepared by reacting zinc hydroxynitrate/zinc oxide mixtures with the general formula [ZnO]_x_[Zn_a_(OH)_b_(NO_3_)_c_·nH_2_O]_y_ (with a = 0.5b + 0.5c) with glutaric acid, while ZnGl-3 was obtained from commercial ZnO and glutaric acid.

X-ray diffraction (XRD) patterns of the prepared materials were measured on a Bruker D8 Advance diffractometer with Cu Kα1 radiation (λ = 1.5418 Å) under 40 kV and 40 mA in the range 5–80° with a step size of 0.02°.

For the synthesis of the ZnGl-1 catalyst, first a [ZnO]_x_[Zn_a_(OH)_b_(NO_3_)_c_·nH_2_O]_y_ precursor was prepared by adding in a single step an aqueous solution of zinc nitrate hexahydrate (Zn(NO_3_)_2_∙6H_2_O, 26.8 g, 0.09 mol) in 30 mL of deionized water to a suspension of zinc oxide (ZnO, 7.3 g, 0.09 mol) in 80 mL of deionized water at room temperature. After stirring the mixture for 1 h (400 rpm), the solid was filtered and washed with water (500 mL) to remove the excess zinc nitrate and then dried in an oven at 90 °C for 40 h. XRD analysis showed that the obtained material (ZnPrec-1) was a mixture of Zn_3_(OH)_4_(NO_3_)_2_ and ZnO (Figure S1). The sample was stored in a desiccator filled with silica gel to prevent possible deterioration of the material caused by reaction with water (moisture). Then, the zinc-based precursor (ZnPrec-1, 2.0 g) and glutaric acid (3.26 g) were added into a 250 mL 3-neck round-bottom flask, to which toluene (150 mL) was added. The round-bottom flask was connected to a condenser and heated to 90 °C, while stirring (500 rpm) for 24 h. After cooling down, the obtained white precipitate was filtered, washed with ethanol (500 mL), and dried at 120 °C overnight. The obtained ZnGl-1 catalyst was characterized by XRD to evaluate the conversion of the Zn precursor and stored in a desiccator filled with silica gel.

For the synthesis of the ZnGl-2 catalyst, the [ZnO]_x_[Zn_a_(OH)_b_(NO_3_)_c_·nH_2_O]_y_ precursor was prepared with a different approach compared to the one used in the synthesis of the ZnGl-1 catalyst. Zn(NO_3_)_2_·6H_2_O (29.8 g) was dissolved in Milli-Q water (1000 mL) to form a 0.1 M solution. An aqueous ammonia solution (13.6 g, 25 wt%) was added dropwise into the zinc nitrate solution while stirring (400 rpm), leading to the formation of a white precipitate. The obtained suspension was aged at room temperature for 2 h while stirring. Then, the solid was filtered and washed with 1 L of water. The obtained white powder was dried at 90 °C for 20 h. XRD analysis showed that the material (ZnPrec-2) was a mixture of Zn_5_(OH)_8_(NO_3_)_2_(H_2_O)_2_ and ZnO (Figure S1). Then, the zinc-based precursor (ZnPrec-2, 2.0 g) was reacted with glutaric acid following the same procedure described for ZnGl-1. The obtained ZnGl-2 catalyst was characterized by XRD and stored in a desiccator filled with silica gel.

For the synthesis of the ZnGl-3 catalyst, commercial ZnO (Sigma Aldrich, Darmstadt, Germany) and glutaric acid were reacted in equimolar amounts following a procedure from the literature with minor modifications [30]. Briefly, ZnO (2.0 g) and glutaric acid (3.26 g) were placed into a 250 mL 3-neck round-bottom flask, then toluene (200 mL) was added. The round-bottom flask was connected to a Dean-Stark trap and heated to 100 °C, while stirring (500 rpm) for 24 h under a N_2_ atmosphere. No water was collected in the Dean Stark trap under these conditions. After cooling down, the formed white precipitate was filtered, washed with ethanol (500 mL), and dried at 120 °C overnight. The obtained ZnGl-3 catalyst was characterized by XRD and stored in a desiccator filled with silica gel.

### 3.2. Synthesis and Characterization of the Polypropylene Carbonate (PPC) Materials

#### 3.2.1. Synthesis of the PPC Materials

**Scheme 1:**
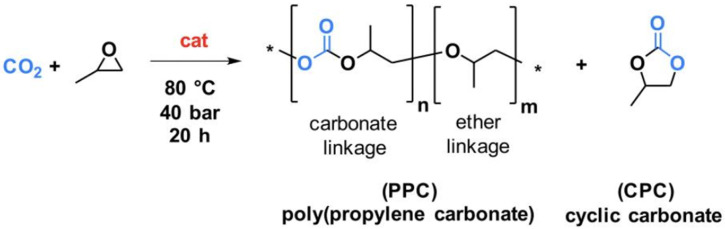
Catalytic synthesis of polypropylene carbonate (PPC) through the copolymerization of CO_2_ with propylene oxide. The * stand for the repetition of the represented molecular unit. In blue is reported the CO_2_ molecules and its trapping PPC and CPC; the red “cat” stands for the catalyst.

Five polypropylene carbonate (PPC) materials were synthesized through the reaction of CO_2_ with propylene oxide (Scheme 1) over a zinc glutarate heterogeneous catalyst (ZnGl-1, ZnGl-2, or ZnGl-3).

The syntheses were conducted in a Parr reactor with a volume of 100 mL under the following reaction conditions: 0.5 g of catalyst (2.56 mmol, assuming the catalyst to be pure zinc glutarate); 50 mL of propylene oxide (PO, 42.95 g, 0.7395 mol); mechanical stirring at 300 rpm; 80 °C, 40 bar CO_2_ (pressure at the start of the reaction, once the reaction temperature was reached); 20 h. Note: drying the catalyst overnight at 120 °C just before the catalytic test is recommended to remove water that may have adsorbed on the material surface in the time passed between the catalyst synthesis and the catalytic test.

For each experiment, 0.5 g of catalyst was added as a solid into the reactor, followed by 50 mL of cold propylene oxide. The reactor was subsequently closed and purged 3 times with 5–10 bar CO_2_. Each time, 1–2 min were waited before depressurizing the reactor to atmospheric pressure. Next, the reactor was pressurized at approximately 35–38 bar CO_2_, and then 30 min were waited to let the system stabilize. During this time, the pressure dropped significantly due to the dissolution of CO_2_ in the epoxide medium. Then, the reactor was repressurized at approximately 38 bar and then heated to 80 °C, which resulted in a final pressure of approximately 40 bar (in case of lower pressure at 80 °C, the reactor was pressurized with CO_2_ to reach 40 bar). The start of the reaction was defined as the moment at which the desired reaction temperature and pressure were reached, after which the stirring was switched on. The reactions were carried out at 300 rpm stirring speed for 20 h. At the end of the reaction, the mechanical stirring and the heating were switched off, and the water-cooling system was turned on to cool down the reactor. Upon reaching room temperature, the reactor was depressurized slowly and gradually. The process of depressurizing the reactor took approximately 2–3 h. After reaching atmospheric pressure, the reactor was opened, and the obtained reaction mixture was poured into a solution of HCl and methanol (0.1 M, 300 mL) to recover the polycarbonate by precipitation. The obtained polymer was washed with methanol (at least three times with 100 mL each) and then dried in a vacuum oven at 55 °C for 3 days. The purified polymer was weighed to determine the isolated yield and productivity. A small portion of the obtained polymer was dissolved in CDCl_3_ and analysed by proton nuclear magnetic resonance spectroscopy (^1^H-NMR) on a Varian Mercury Plus 400 MHz (Varian Inc., Palo Alto, CA, USA) or Agilent MR 400 MHz (Agilent Tchnologies, Inc., Santa Clara, CA, USA) apparatus to determine the yields of polypropylene carbonate (including the fraction of carbonate and ether linkages in the polymer) and of cyclic propylene carbonate (CPC).

In order to decrease the CPC fraction in the final polymer product, the purification procedure was optimized: pouring slowly the reaction mixture into the HCl/MeOH solution and cutting the purified polymer into small pieces and sonicating it repeatedly in MeOH were found to lead to a polycarbonate product with lower CPC content.

Three PPC materials were obtained using ZnGl-1 as the catalyst (labeled PPC100, PPC101, PPC102), one was synthesized using ZnGl-2 as the catalyst (PPC103), and the last one was prepared using ZnGl-3 as the catalyst (PPC104) (see Table 1).

#### 3.2.2. Characterization of the Molecular Weights of the PPCs by GPC

The molecular weights (M_w_ and M_n_) of the purified polycarbonates were determined by Gel Permeation Chromatography (GPC), using tetrahydrofuran as solvent and toluene as internal standard, on an Agilent HPLC 1100 (Agilent Tchnologies, Inc., Santa Clara, CA, USA) system equipped with three MIXED-E columns (length: 300 mm, i.d.: 7.5 mm) in series and a GBC LC 1240 refractive index detector (RID). The molecular weights were calculated based on a calibration with polystyrene.

#### 3.2.3. Specimen Preparation

The output of the material synthesis may be described as a highly viscous compound that requires adequate processing to provide specimens for mechanical characterization. The production process was properly set up to create specimens with a regular shape, that may be easily measured and gripped in mechanical tests, and with no defects (e.g., voids, surface scratches) nor irregularities (e.g., variable thickness). With these requirements as a target, PPC was first shaped into films by means of a compression molding machine (P 200 E, COLLIN Lab & Pilot Solutions GmbH, Maitenbeth, Germany) and then cut into regular strips for mechanical tests.

For compression molding, a few small pieces of material (typically 3–4 g) were sandwiched between two Teflon^TM^ sheets and subjected to a thermomechanical program consisting of three steps: (i) heating at a given temperature (T_mold_ = 150–165 °C, see Table 1) and applying a moderate pressure (5 bar) for 200 s to ensure the melting of the material by contact with the heating plates; (ii) hot pressing the sample by maintaining the same temperature while applying a higher pressure (30 bar) for 40 s to uniformly distribute the heated material between the plates; (iii) cooling the sample at room temperature under the applied pressure for 300 s to fix this shape, obtaining sheets with a thickness between 0.1 and 0.3 mm. Finally, the Teflon^TM^ sheets were peeled off from the PPC film.

#### 3.2.4. Thermal Properties Characterization

The thermal properties of the materials obtained were characterized in terms of calorimetric response, evaluated by means of DSC (Differential Scanning Calorimetry) tests, and of degradation temperatures, measured by means of TGA (Thermogravimetric Analysis).

DSC tests were carried out by means of a DSC Q100 (TA Instruments, New Castle, DE, USA) calorimeter, under a nitrogen atmosphere. Samples of about 5–10 mg were cut from the specimens and subjected to the following thermal history: (i) heating from −50 °C to 150 °C at 10 °C/min; (ii) cooling from 150 °C to −50 °C at 10 °C/min; (iii) heating from −50 °C to 150 °C at 10 °C/min. Glass transition temperature values were measured on the second heating scan.

TGA was performed by means of a TGA Q500 (TA Instruments, New Castle, DE, USA) analyzer, under an air atmosphere. A small amount of the raw material (5–10 mg) was heated from room temperature (ca. 23 °C) to 600 °C with a dynamic heating rate (Hi-Res^TM^ Dynamic Rate mode of the instrument, which adjusts the heating rate according to changes in the decomposition rate of the sample).

#### 3.2.5. Mechanical Properties Characterization

As a basic mechanical characterization of the materials, tensile tests were performed on rectangular strips cut from the films. The specimens were 80 mm long and 10 mm wide, while their thickness values varied according to those of the films obtained by compression molding (around 0.1–0.3 mm).

Tensile tests were carried out at room temperature (ca. 23 °C) by means of an electromechanical dynamometer (Instron 3366, Norwood, MA, USA), using a load cell of 500 N and clamping the specimens with an initial gauge length (L_i_) of 60 mm. Crosshead displacement rates varying between 10 mm/min and 500 mm/min were explored in order to investigate the effect of the strain rate on the material response.

From stress (σ) vs. strain (ε) curves, the following mechanical properties were obtained: Young’s modulus (E), evaluated in the linear part of stress-strain curves; failure stress (σ_f_), measured in correspondence of the first peak or knee of the curves; ultimate tensile strain (ε_u_), that is the strain at break. The ability of the materials to recover the applied strain was also tracked, measuring the amount of recovered strain at different time points after the end of the test (t = 1 min, 5 min, 1 h, 3 days), according to the following equation:

Recovery [%] = [1 − (L(t) − L_i_)/L(0)]·100 (1)

where L(t) is the gauge length at time point t (measured on juxtaposed specimen stumps) and L(0) is the gauge length at the end of the test (calculated as ε_u_·L_i_).

#### 3.2.6. Creep Compliance Measurement

Creep compliance measurements were carried out by means of a dynamic mechanical analyzer, DMA Q800 (TA Instruments, New Castle, DE, USA), under tensile conditions, on small rectangular specimens cut from PPC films (width: about 5 mm; gauge length: about 15 mm). Creep experiments were performed with a multi-temperature procedure, consisting of subjecting a single specimen to multiple steps of isothermal creep under a fixed stress equal to 0.2 MPa, each step carried out for about 10 min at a different temperature. More specifically, the specimen was first cooled down to −30 °C and then subjected to isothermal steps at increasing temperature values, equally spaced by 5 °C, up to the maximum temperature allowed before the material approaches flow, when the specimen rapidly elongates beyond the maximum length the machine can measure. The aim of these tests is to obtain a full master curve representation of the creep compliance as a function of time, by applying the time-temperature superposition principle to the isothermal creep results obtained at various temperatures.

The choice of the creep modality, instead of the most employed multi-frequency DMA tests, is due to difficulties in collecting data under the typical DMA conditions. In fact, under these conditions, the frequency sweeps were often incomplete, and the tests usually stopped at about 24 °C, close to the material T_g_, around which data collection was inhibited because of the relevant relaxation process occurring during the glass transition. Thanks to the creep modality, it was possible to reach higher temperatures (around 48 °C, with slight differences from sample to sample), approaching the region of flow regime, when the creep compliance increases indefinitely.

#### 3.2.7. Characterization of the Self-Healing Behavior

The self-healing behavior of the PPC materials was characterized by two types of tests, namely single-lap shear tests and T-peel tests.

##### Single-Lap Shear Tests

**Figure 9:**
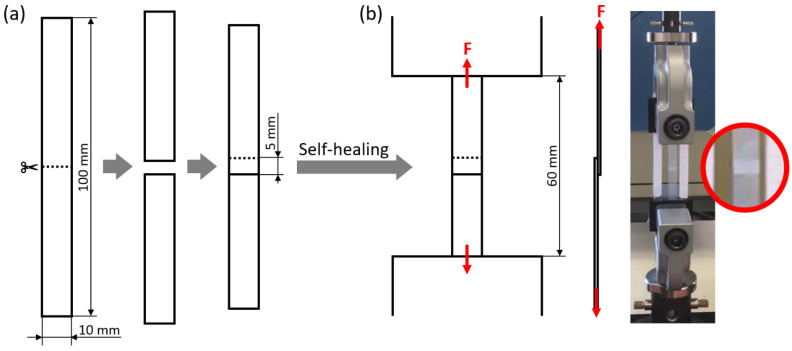
(**a**) Single-lap joint specimen preparation by cutting a rectangular strip in half and overlapping the new extremities; (**b**) tensile testing of the single-lap joint specimen, with close-up on the overlapped region.

Single-lap shear tests were used to evaluate the self-healing ability of rectangular specimens in a single-lap joint configuration. In order to obtain single-lap joint specimens, rectangular strips (width: 10 mm; overall length: 100 mm) were first cut into two halves, and then joined again by overlapping the specimen halves over a 5 mm-long region, as illustrated in Figure 9a. The self-healing of the specimens was promoted under various conditions (see Table 4): at room temperature (T_room_: ca. 23 °C), for times between 10 min and 1 week, by simply maintaining the two halves in contact (pressure on the overlapped region: 0 MPa) or by applying a load (1.6-kg steel block applied on an overlapped region of 10 mm × 5 mm; pressure on the overlapped region: about 0.3 MPa); at 50 °C, for times between 10 min and 1 day, with a pressure of 0 MPa or 0.3 MPa on the overlapped region. The samples were finally tested under tensile conditions in an electromechanical dynamometer (Instron 3366, Norwood, MA, USA) with a gauge length of 60 mm, at a crosshead displacement rate of 50 mm/min, and at room temperature, as illustrated in Figure 9b. For each test, it was evaluated whether the specimen breaks by adhesive failure (detachment of the two overlapped surfaces) or undergoes elongation up to large strains, which would indicate the restoring of a level of structural integrity allowing it to fully withstand tensile stress without undergoing failure in the joint region. By carrying out the tests at different times after overlapping the specimen halves, it was also possible to quantify the time required to achieve such a level of self-healing. In addition, for specimens undergoing adhesive failure, the adhesion strength of the two overlapping surfaces was evaluated as follows:

τ_adh_ [MPa] = F_max_/A_over_ (2)

where F_max_ [N] is the maximum force measured at failure, while A_over_ [mm^2^] is the area of the overlapped region.

##### T-Peel Tests

**Figure 10:**
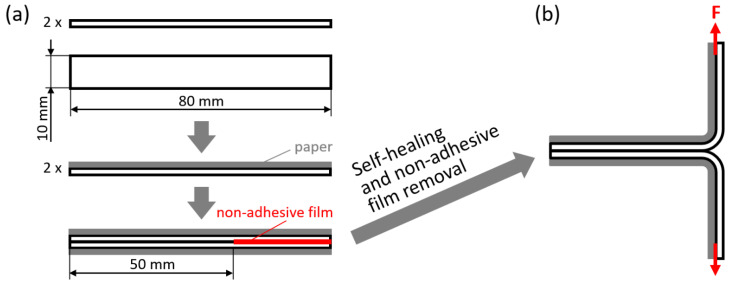
(**a**) Specimen preparation for T-peel tests, by first taping a paper substrate to two rectangular strips of PPC and then overlapping the strips with the application of a non-adhesive film at one extremity; (**b**) T-peel testing of the obtained specimen.

The self-healing abilities were also evaluated in a T-peel configuration, with the specific aim of evaluating the adhesion achieved between overlapped PPC surfaces after self-healing. The specimens were obtained as shown in Figure 10a: two rectangular strips (width: 10 mm; overall length: 80 mm) were first attached to a paper substrate on one side (by means of double-sided tape), and then placed in mutual direct contact on the other side over a 50 mm-long region, separating them by a non-adhesive Teflon^TM^ film for the remaining 30 mm length. Also in this case, self-healing in the overlapping region was promoted under various conditions (see Table 4): at room temperature (T_room_, ca. 23 °C), for times between 10 min and 1 week, with a pressure of 0 MPa or 0.03 MPa (1.6 kg steel block applied on an overlapped region of 10 mm × 50 mm); at 50 °C, for times between 10 min and 1 day, with a pressure of 0 MPa or 0.03 MPa. Finally, the two non-adhered extremities were clamped in the electromechanical dynamometer (Instron Mod. 3366, Norwood, MA, USA) and pulled apart with a crosshead displacement rate of 50 mm/min at room temperature, as illustrated in Figure 10b. Importantly, the paper substrate provided structural stiffness to the clamped extremities, thus avoiding their deformation and allowing us to consider these experiments as peeling tests in which the load measured by the dynamometer corresponds to the force required to separate the two adhered extremities. The force necessary to peel a unit-width specimen was evaluated as follows:

F_peel_ [N/cm] = F_max,ave_/w (3)

where F_max,ave_ [N] is the average force measured in correspondence with the 10 highest peaks in the “stick-slip” plateau, while w [cm] is the specimen width.

## 4. Conclusions

Carbon capture and utilization is recognized as a virtuous approach to reduce the environmental impact of CO_2_ industrial emissions, and was achieved here by utilizing carbon dioxide as a monomer for the production of PPC, through the copolymerization with propylene oxide over zinc glutarate catalysts.

The prepared PPC differed in terms of molecular weight, fraction of ether linkages, and content of cyclic propylene carbonate, with the last feature showing the strongest correlation with the observed differences in glass transition temperature (with higher CPC content leading to lower T_g_). A thorough characterization showed that the produced PPCs exhibit sufficient strength and stiffness to compete with common fossil-based thermoplastics, while displaying great flexibility and remarkably high elongation at break (up to 860%) in the absence of added plasticizers, thus appearing promising for application in packaging and other flexible products. Moreover, the PPC materials were able to quickly and fully recover deformation after being stretched up to exceptionally high strains, even above 700%. Variations in these mechanical properties could be associated with differences in the thermal properties, with a lower glass transition temperature inducing a softening effect and an increase in ductility in the materials. In addition, a significant effect of the strain rate on the viscoelastic mechanical response of PPC was also highlighted, suggesting the possibility of tuning the stiffness and strength of the material for a specific application related to a given strain rate.

Finally, PPC was found to have self-healing capabilities, through which the materials can recreate a strong seal when two surfaces are put together. Self-healing at room temperature was slow, but a moderate increase in temperature, in particular above T_g_, greatly promoted the process thanks to the increase in molecular mobility. At 50 °C (i.e., about 20–25 °C above the T_g_), the time needed for adequate repair of the material varied from just a few minutes to maximum 1 day, depending on the use of pressure, on the severity of the test type, and on the characteristic T_g_ and flow time of the specific PPC. In order to achieve sufficient self-healing to withstand shear forces, as revealed by single lap shear tests, the exposure to temperatures above T_g_ for a few minutes may suffice, whereas longer treatment times were required to obtain stronger joints, capable of withstanding peeling. In particular, it was shown that such self-healing times scale well with the flow times associated with the specific self-healing temperature, since flow-related processes such as re-entanglement are believed to be at the basis of the creation of a more intimate connection of the overlapped portion. Notably, for each of the examined materials, the relative master curve can help estimate the time necessary for proper adhesion of two PPC surfaces at any temperature.

Thanks to their processability by means of typical processes for thermoplastics and to the promising qualities reported in this study, the synthesized PPCs could be manufactured in various forms and applied in several industrial fields. Their high flexibility, remarkable strain recovery after stretching, and self-healing behavior can be especially appealing in packaging applications such as shrink films for wrapping, self-welding tapes, and long-lasting or even reusable packages.

## Figures and Tables

**Table 1:** Physicochemical and thermal properties of the PPC materials: composition; content of ether linkages (%); weight-average molecular weight (M_w_); number-average molecular weight (M_n_); polydispersity index (D = M_w_/M_n_); molding temperature (T_mold_); glass transition temperature (T_g_); temperature associated with the main thermal degradation process (T_deg_).

Material Code	Catalyst	Composition [wt%]		Ether Linkage %	M_w_ [g/mol]	M_n_ [g/mol]	D	T_mold_ [°C]	T_g_ [°C]	T_deg_ [°C]
		PPC	CPC							
PPC100	ZnGl-1	≥99.5	≤0.5	5.7	44.9·10^3^	14.3·10^3^	3.1	150	30.0	195
PPC101	ZnGl-1	98.6	1.4	4.9	98.0·10^3^	36.7·10^3^	2.7	155	28.4	212
PPC102	ZnGl-1	97.8	2.2	5.3	122.6·10^3^	35.9·10^3^	3.4	155	25.8	212
PPC103	ZnGl-2	≥99.5	≤ 0.5	6.1	353.2·10^3^	58.8·10^3^	6.0	165	30.1	246
PPC104	ZnGl-3	97.4	2.6	6.5	299.0·10^3^	49.1·10^3^	6.1	155	25.6	224

**Table 2:** Young’s modulus, tensile strength, and elongation at break of various PPCs.

Reference	Young’s Modulus [MPa]	Tensile Strength [MPa]	Elongation at Break [%]
This work	45–2100	1–30	2–860
Thorat et al. [35]	212	9	8
Hsu and Tan [36]	1353	14.7	203.1
Luinstra [37]	200–1400	7–30	–
Trofimchuk et al. [38]	90–2120	1–41	4–940
Du et al. [39]	830	21.5	330
Hao et al. [3]	1080	37.1	7.3
Muthuraj and Mekonnen [19]	680–2000	15–21.5	255–330
Ye et al. [18]	400–1800	11–40	15–600
Bora et al. [11]	–	33–35	450–520

**Table 3:** Characteristic retardation times (t_ret_) and flow times (t_flow_) of the PPC materials at two reference temperatures: T_0_ = 20 °C and T_0_ = 50 °C.

System	T_0_ = 20 °C		T_0_ = 50 °C	
	t_ret_	t_flow_	t_ret_	t_flow_
PPC100	1.6·10^3^ min (1·1 d)	8.5·10^4^ min (60 d)	2.5·10^−3^ min (0.15 s)	1.3·10^−1^ min (8 s)
PPC101	1.7·10^2^ min (0.12 d)	8.4·10^5^ min (600 d)	1.2·10^−3^ min (0.07 s)	6.0·10^0^ min (400 s)
PPC102	3.4·10^0^ min (0.002 d)	1.0·10^4^ min (7 d)	8.9·10^−5^ min (0.005 s)	2.6·10^−1^ min (16 s)
PPC103	1.2·10^3^ min (0.8 d)	1.4·10^7^ min (10000 d)	1.8·10^−3^ min (0.11 s)	2.0·10^1^ min (1200 s)
PPC104	1.1·10^2^ min (0.08 d)	2.7·10^5^ min (200 d)	4.3·10^−4^ min (0.03 s)	1.1·10^0^ min (60 s)

**Table 4:** Temperature, time, and pressure conditions at which self-healing of the specimens was promoted before the single-lap shear tests and T-peel tests.

Type of Test	Adhesion Temperature	Adhesion Time	Adhesion Pressure
Single-lap shear test	T_room_ (ca. 23 °C)	10 min; 2 h; 1 d; 1 w	0 MPa; 0.3 MPa
Single-lap shear test	50 °C	10 min; 2 h; 1 d	0 MPa; 0.3 MPa
T-peel test	T_room_ (ca. 23 °C)	10 min; 2 h; 1 d; 1 w	0 MPa; 0.03 MPa
T-peel test	50 °C	10 min; 2 h; 1 d	0 MPa; 0.03 MPa

## Data Availability

The original contributions presented in this study are included in the article/Supplementary Materials. Further inquiries can be directed to the corresponding author(s).

## Supplementary Materials

The following supporting information can be downloaded at: https://www.mdpi.com/article/10.3390/ijms26083878/s1.
