# Supplementary material for: CO2-Based Polypropylene Carbonates with High-Stretch and Self-Healing Properties
Source: Int J Mol Sci. 2025 Apr 19;26(8):3878. doi: 10.3390/ijms26083878 (PMC12027896; doi:10.3390/ijms26083878)
Supplement: Supplementary file 1 [file ijms-26-03878-s001.zip › ijms-3549540-supplementary.pdf]

CO_2-Based Polypropylene Carbonates with High-Stretch and Self-Healing Properties

Chiara Pasini, Stefano Pandini, Francesca Milocco, Jing Chen, Zhenchen Tang, Paolo P. Pescarmona and Luciana Sartore

Supporting Information

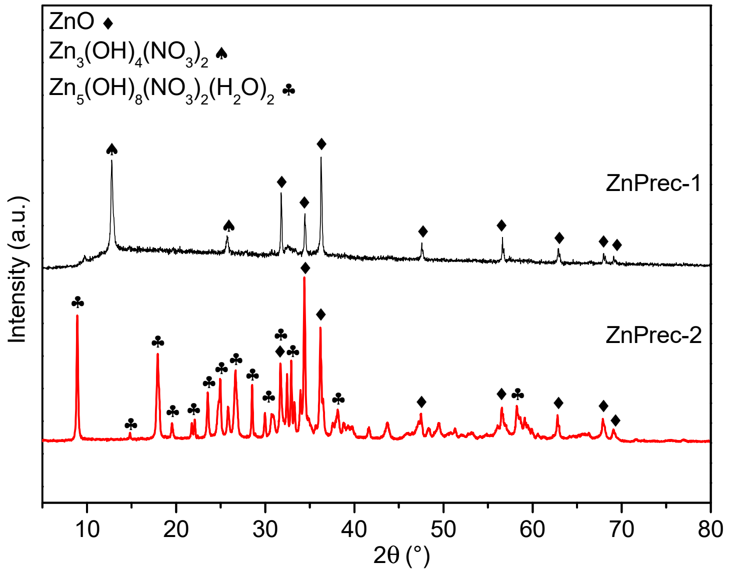

Figure S1: XRD patterns of the zinc precursors used to prepare the zinc glutarate catalysts ZnGl-1 and ZnGl-2.

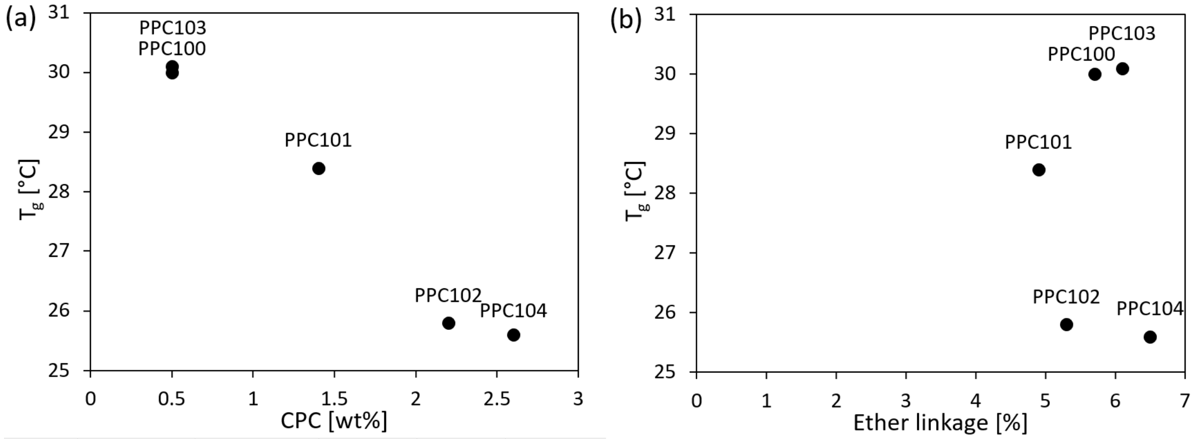

Figure S2: Glass transition temperature (T_g) of the polypropylene carbonate (PPC) materials as a function of the cyclic propylene carbonate (CPC) content (a) and of the percentage of ether linkages (b).

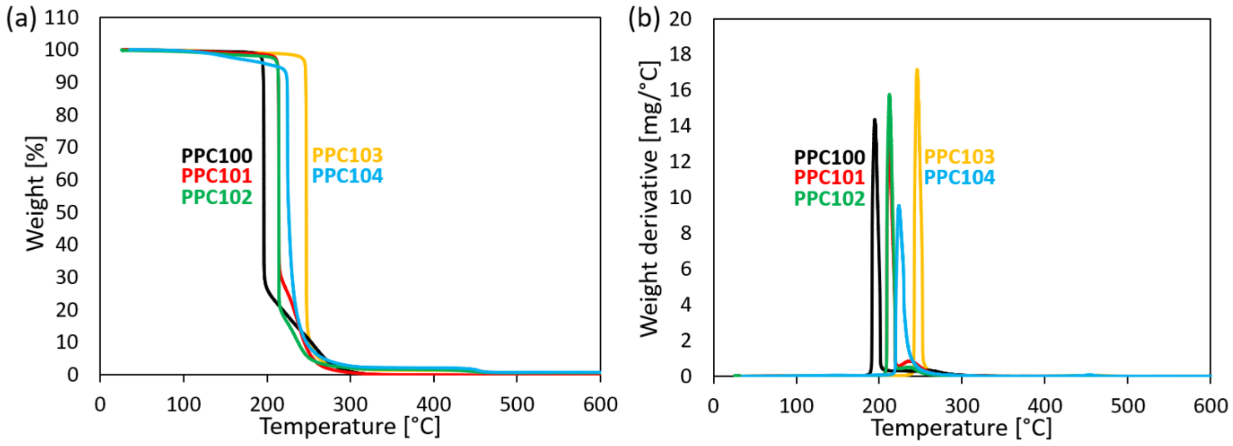

Figure S3: TGA traces of the PPCs: (a) weight and (b) weight derivative as a function of temperature.

Table S1: Comparison between the flow times obtained in creep tests for T_0 = 20 °C or 50 °C and the times required for effective self-healing according to single-lap shear tests and T-peel tests after joining at T_{room} or 50 °C, and in either absence or presence of an external pressure (w/o p; with p).

**Temperature**	**20 °C / T_{room}**			**50 °C**				
**Time for**	**Flow**	**Self-healing**		**Flow**	**Self-healing**			
**Test**	**Creep**	**Single-lap (with p)**	**T-peel (with p)**	**Creep**	**Single-lap (w/o p)**	**T-peel (w/o p)**	**Single-lap (with p)**	**T-peel (with p)**
**PPC100**	60 d	1 to 7 d	> 7 d	0.13 min	< 10 min	10 min to 2 h	< 10 min	< 10 min
**PPC101**	600 d	> 7 d	> 7 d	6.0 min	< 10 min	2 h to 1 d	< 10 min	10 min to 2 h
**PPC102**	7 d	1 to 7 d	> 7 d	0.26 min	< 10 min	2 h to 1 d	< 10 min	10 min to 2 h
**PPC103**	10^4 d	> 7 d	> 7 d	20 min	< 10 min	> 1 d	10 min to 2 h	2 h to 1 d
**PPC104**	200 d	> 7 d	> 7 d	1.1 min	< 10 min	2 h to 1 d	< 10 min	2 h to 1 d
